# Formation of broadband antireflective and superhydrophilic subwavelength structures on fused silica using one-step self-masking reactive ion etching

**DOI:** 10.1038/srep13023

**Published:** 2015-08-13

**Authors:** Xin Ye, Xiaodong Jiang, Jin Huang, Feng Geng, Laixi Sun, Xiaotao Zu, Weidong Wu, Wanguo Zheng

**Affiliations:** 1 Research Center of Laser Fusion, China Academy of Engineering Physics, Mianyang, 621900 (P.R. China); 2 Science and Technology on Plasma Physics Laboratory, Research Center of Laser Fusion, China Academy of Engineering Physics, Mianyang, 621900 (P.R. China); 3 School of Physical Electronics, University of Electronic Science and Technology of China, Chengdu, 610054 (P.R. China)

## Abstract

Fused silica subwavelength structures (SWSs) with an average period of ~100 nm were fabricated using an efficient approach based on one-step self-masking reactive ion etching. The subwavelength structures exhibited excellent broadband antireflection properties from the ultraviolet to near-infrared wavelength range. These properties are attributable to the graded refractive index for the transition from air to the fused silica substrate that is produced by the ideal nanocone subwavelength structures. The transmittance in the 400–700 nm range increased from approximately 93% for the polished fused silica to greater than 99% for the subwavelength structure layer on fused silica. Achieving broadband antireflection in the visible and near-infrared wavelength range by appropriate matching of the SWS heights on the front and back sides of the fused silica is a novel strategy. The measured antireflection properties are consistent with the results of theoretical analysis using a finite-difference time-domain (FDTD) method. This method is also applicable to diffraction grating fabrication. Moreover, the surface of the subwavelength structures exhibits significant superhydrophilic properties.

Antireflection (AR) technology is indispensable for improving the performance of materials for applications such as light emitting diodes^1^, flat-panel displays, solar cells^2^, and optical sensors^3^. AR technology is often required to suppress or eliminate reflection while increasing the transmittance of transparent optical elements. One approach for creating an antireflective coating is to prepare multilayer or porous films on the surface of optical elements^4^,^5^. However, inherent coating limitations, such as adhesiveness, stability, low laser damage thresholds, and thermal expansion mismatch, are often encountered^6^. Inspired by biological compound eyes, the fabrication of artificial moth eye structures, also referred to as subwavelength structures (SWSs), on the surfaces of devices has attracted significant interest as a powerful method for obtaining AR properties^7^,^8^,^9^. SWSs can overcome the abovementioned problems of film coatings because the SWSs are prepared from the same material as the substrate^10^. In the past decade, extensive research on obtaining SWSs with broadband AR performance has examined anodic aluminum oxide^11^,^12^, magnetron sputter deposition of metallic nanoparticles^13^, interference lithography^14^, electron-beam lithography^15^, and thermally dewetted metallic nanoparticles^16^ as reactive ion etching (RIE) masks. However, all of these nanofabrication methods have drawbacks, such as the use of multiple expensive steps and time-consuming procedures. These complications have prevented large-scale production, which is required for practical applications.

Well-ordered monolayer particle arrays can also be used as etching masks. This approach is based on a simple and scalable self-assembly technique for fabricating SWSs on a planar or grated silicon substrate surface^17^,^18^. However, the development of new fabrication approaches, particularly maskless, low-cost, one-step fabrication methods for AR surfaces, is a key issue in the production of optical devices.

An one-step sputtered aluminum micro-etch-masking strategy was recently reported for fabricating fused silica grass using plasma-etching processes^19^. However, contamination by aluminum impurities during SWSs fabrication resulted in a low laser damage threshold^20^,^21^.

Polymers can be deposited on the sample surface during RIE under certain conditions^22^. These polymers can act as micro-etching masks, resulting in the formation of RIE grass structures with stochastic nanocones. This method has been developed into a one-step, maskless, and inexpensive approach for fabricating SWSs on silicon substrates^23^,^24^. Fused silica is one of the most widely used materials for optical and optoelectronic applications, such as optical windows, imaging systems, and high-power laser devices^25^,^26^. However, this method has not been widely employed for producing SWSs on a planar or non-planar fused silica substrate.

In this paper, we report a one-step, scalable approach for the maskless fabrication of planar fused silica SWSs and fused silica grating SWSs using RIE under specific conditions. The fabricated SWS surfaces have excellent broadband AR properties in the ultraviolet to near-infrared wavelength region. Multifunctional AR surfaces with antifogging properties have attracted significant recent attention^27^. This added functionality can significantly extend the use of AR surfaces for applications such as goggles, vehicle windshields, solar cells, and optical devices. Accordingly, we also demonstrate that fabricated SWSs on fused silica exhibit superhydrophilic properties and therefore have potential antifogging applications.

## Results

**Figure 1:**
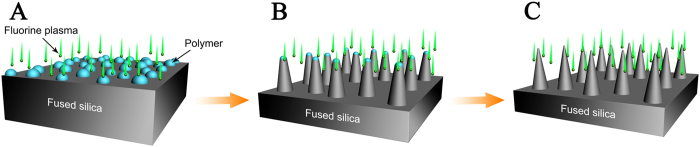
Schematic illustration of the SWS preparation process.

Polymer nanodots formed on the substrate surface during the RIE process under certain plasma conditions. These polymer nanodots can be considered random micro-etch masks on the fused silica surface (Fig. 1A). A fused silica cone-like profile with the polymer nanodot tips was formed using reactive radical etching of the substrate surface (Fig. 1B). The polymer nanodots were etched during the RIE process, but the etching speed was much slower than the fused silica. In addition, polymers were deposited on the surface throughout the etching process. The surface peaks received more deposited polymer than the valleys. Furthermore, the polymer nanodots formed on the nanocone tips induced greater polymer deposition. Therefore, the height of the fused silica nanocones could be controlled by altering the etching conditions. Owing to isotropic etching during the RIE process, tapered sidewalls of the silica SWS formed on the fused silica substrate. Random fused silica SWSs with a tapered profile and high aspect ratio were obtained, as shown schematically in Fig. 1. The samples were then cleaned using O_2_ plasma for 5 min to remove the polymer residue.

**Figure 2:**
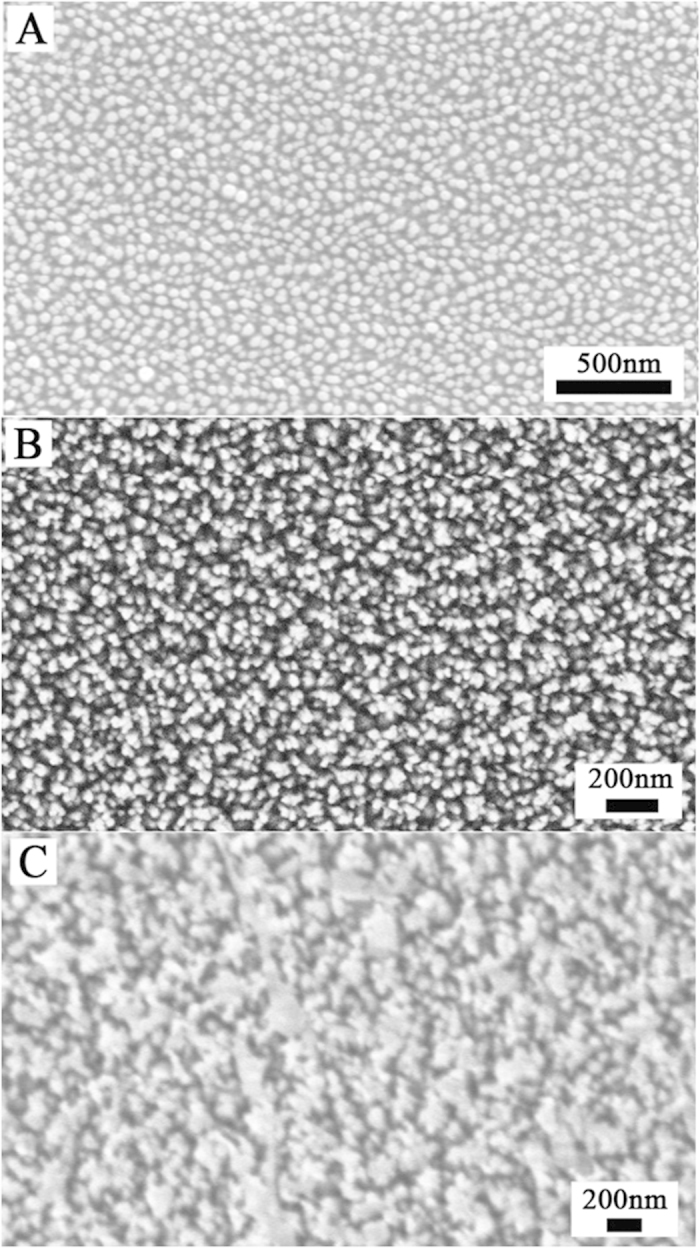
SEM images of the top view of the fused silica SWSs prepared at different CHF_3_ flow rates: (**A**) 25 sccm, (**B**) 30 sccm, (**C**) 40 sccm.

The self-masking mechanism is primarily due to polymer deposition. To determine the elemental composition of the by-products during plasma etching, X-ray photoelectron spectroscopy (XPS) analysis was performed. XPS provides information about the elemental composition of a surface and its electron configuration. Silicon-oxygen (fused silica), carbon-oxygen (CO_2_), and carbon-fluorine (polymers) bonds were detected on the self-masking RIE sample surface. However, carbon-fluorine (polymer) bonds were not observed on the conventional RIE sample surface, as shown in Figure S1. Polymer deposition formation has been found to be well correlated with high concentrations of CF and CF_2_ radicals^28^. The SWSs morphology could be varied by controlling the CHF_3_ flow rate and etching time of the RIE process. Figure 2 shows the morphologies of SWSs etched under various CHF_3_ flow rates. The corresponding processing parameters are provided in Table 1. As shown in Fig. 2(A) (sample S1, average period ~80 nm), Fig. 2(B) (sample S2, average period ~100 nm), and Fig. 2(C) (sample S3, average period ~240 nm), the average period of the SWSs gradually increased with increasing CHF_3_ flow rate.

**Figure 3:**
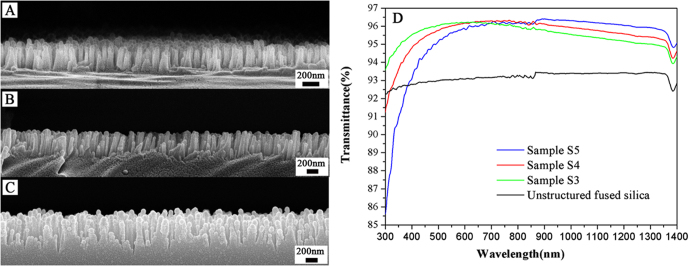
Morphology of the fused silica SWSs prepared using different etching times: (**A**) 20 min, (**B**) 30 min, (**C**) 40 min. (**D**) Transmission of the fused silica SWS samples after etching times of 20 min (green line), 30 min (red line), and 40 min (blue line).

The SWS height was controlled by varying the etching time. As shown in Fig. 3(A), the SWS height after 20 min of etching was approximately 290 nm. By extending the etching time to 30 min, the SWS height increased to 410 nm (Fig. 3B). After a further increase in the etching time to 40 min, the SWS height was approximately 540 nm. AR behavior was characterized by measuring transmission. As shown in Fig. 3(D), the transmittance for the 480–800 nm wavelength range increased from approximately 93% for the fused silica substrate (black line) to greater than 96% for the 20-min single-side etched sample (green line). The other two samples also exhibited strong AR behavior at the measured wavelengths. For instance, the transmittance of the S4 sample increased to greater than 96% (30 min etched, red line) for the 550–970 nm wavelength range. The transmittance of the 40-min etched sample (sample S5, blue line) was greater than 96% for the 650–1250 nm wavelength range. The SWSs with greater height exhibited improved broadband AR performance. As the etching time increased, the height of the SWSs gradually increased, whereas the average SWS period increased because the adjacent structures aggregated together. In addition, as the SWS height increased, the transmittance in the short wavelength range decreased, primarily due to light scattering on the surface.

**Figure 4:**
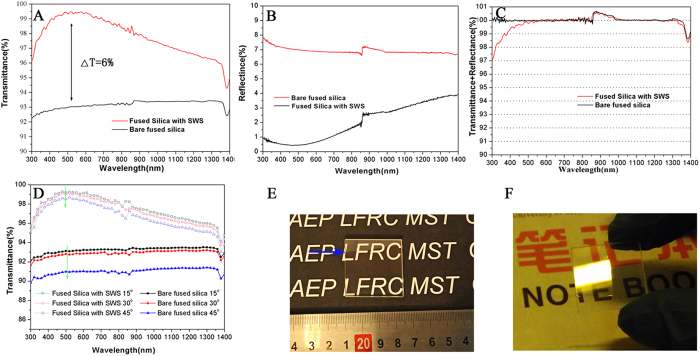
(**A**) Transmittance of the fused silica double-sided SWS and the bare fused silica. (**B**) Reflectance of the fused silica double-sided SWS and the bare fused silica. (**C**) The sum of the measured transmittance and reflectance of the fused silica double-sided SWS. (**D**) The angle-dependent transmission spectra for the SWS and bare fused silica at 15°, 30° and 45°. Note the OH^−^ absorption at approximately 1380 nm and the detector change at approximately 860 nm. (**E**) Photograph of the SWS under sun light illumination. The boundary between the coated (bottom of the substrate) and uncoated (top of the substrate) area of the SWS is indicated by the blue arrow. (**F**) Photograph of a fluorescent light reflected on the SWS surface that was fabricated on one half of the fused silica substrate. Figures (**E**,**F**) were obtained by X. Ye and L. Sun.

Using the RIE procedure described above, a double-sided SWS was fabricated using the S3 condition from the broadband AR performance study. In the double-sided SWS, the morphologies of the two sides were nearly identical because the fabrication process was identical for both sides (Supplementary Information Figure S2). Figure 4(A) presents the optical transmission spectra of the fused silica with the double-sided SWS (red line) and the bare fused silica (black line) with a normal incident source. The transmittance of the SWS sample was significantly improved for a wide range of wavelengths (300–1400 nm) compared with the bare fused silica. The maximum transmittance of the double-sided surfaces of the SWS at 500 nm was approximately 99.5%, ~6% higher than the transmittance of the bare fused silica. Moreover, the double-sided surfaces of the SWS exhibited transmittance of greater than 99% for the 400–700 nm wavelength range. The hemispherical total reflectance (diffuse + specular) as a function of wavelength for the fused silica with the double-sided SWS (black line) and without the SWS (red line) using the integrating sphere is presented in Fig. 4B. The average hemispherical total reflectance decreased to <1% for the 300–700 nm wavelength range, with an average hemispherical total reflectance of <0.5% for the 410–550 nm wavelength range. The specular and diffused scattering components are presented in Figure S3 (A). As shown in Fig. 4(C), the sum of the transmittance and reflectance was less than 100% in the short wavelength region for the fused silica SWS and approximately 100% at all measured wavelengths for the bare fused silica substrate. This observation will be discussed later. The angle-dependent transmittance of the surfaces of the SWS was evaluated at different incident angles from 15° to 45°. As shown in Fig. 4(D), the fused silica SWS had a nearly constant average transmittance of approximately 99% for an incidence angle up to 30°. The maximum transmittance of the double-sided surfaces of the SWS was approximately 98.4% for an incidence angle up to 45°. However, the transmittance of the fused silica with the double-sided SWS was less dependent on the incidence angle of light than was the bare fused silica substrate. The fused silica with the double-sided SWS exhibited a ~1% decrease in the transmittance value for incidence angles of 0° to 45°, a smaller decrease than that observed for the bare fused silica (>2%). This difference occurs because the effective refractive index profile from air to the fused silica substrate changes slowly with an increase in the incidence angles. As indicated by the black line in Fig. 4D, all the transmittance data for the double-sided SWS were larger than those of the bare fused silica substrate, confirming the excellent large field of view AR characteristics of the double-sided SWS.

The decreased reflectivity in the visible light region of the SWS portion of the fused silica substrate is evident in Fig. 4(E,F). In Fig. 4(E), the words that are covered by the fused silica SWS are clearly visible, demonstrating the low reflectance of the SWS portion of the fused silica surface below the line indicated by the blue arrow, which marks the borderline between the SWS and non-SWS portions of the fused silica surface. However, the non-SWS portion of the fused silica surface above the borderline exhibits intense sunlight reflectivity. Figure 4(F) presents the comparison of the SWS and the non-SWS halves of the substrate under fluorescent light illumination. The non-SWS half of the fused silica reflects the image of the fluorescent light, but the SWS half provides a clear vision.

## Discussion

In the present study, Fresnel reflection of incident light occurred at the interface between fused silica and air due to the large refractive index discontinuity. According to effective medium theory, the SWSs on the substrate surface are considered an inserted layer. The gradual change in the refractive index from air to the silica substrate dramatically suppressed the Fresnel reflection from the fused silica substrate. For an ideal SWS with a broadband AR, the effective refraction index *n*_*eff*_ should gradually increase from *n*_*air*_ to *n*_*silica*_ from the external medium to the fused silica substrate.

Rigorous coupled wave analysis (RCWA)^29^ and finite-difference time-domain (FDTD)^30^ methods have been used to accurately fine-tune the analysis of ideal anti-reflective interfaces. Because technological improvements have made 3D-FDTD modeling faster and easier, the experimental data were analyzed by theoretical calculations using the 3D-FDTD method. The theoretical calculations provided insight into the reflectance of regular nanostructures with a hexagonal arrangement. The shapes of the nanostructures were described using the profile functions with the parameters D and H. D is the diameter of the nanostructures, which was derived from an average period of 100 nm. H is the height of the nanostructures (the detailed setup is explained in the Materials and methods section). The reflectance of the cone, paraboloid, and truncated cone were calculated. For the truncated cone, the calculations were performed for shapes with apex diameters of 25% and 50% of the base. For the paraboloid, the apex radius of the curvature was set to 10 nm. The substrate (Fused silica Corning 7980, Corning Inc., Corning, USA) was taken into account in this calculation.

**Figure 5:**
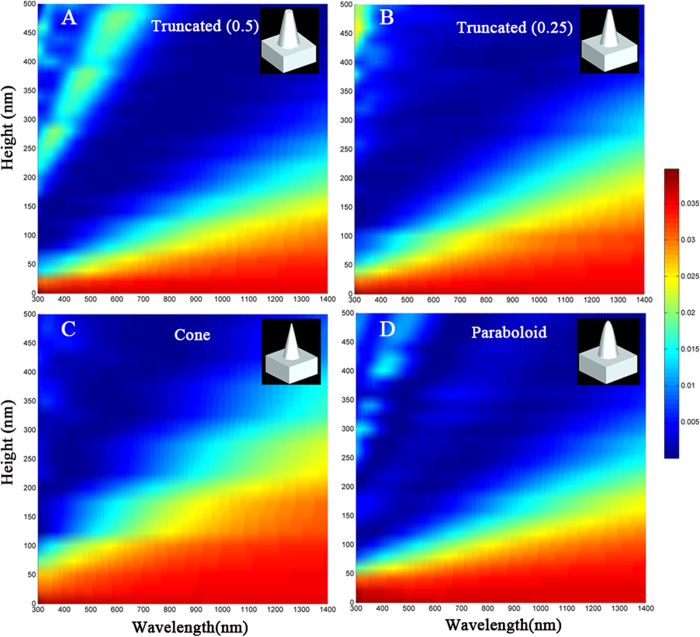
Two-dimensional contour plots of the calculated reflectance for various SWS shapes according to height and the wavelength of light: (**A**) truncated cone (50%), (**B**) truncated cone (25%), (**C**) cone, (**D**) paraboloid profiles.

Figure 5 presents the contour plots of the calculated reflectance result variations as a function of wavelength for the height of the SWS with (A) truncated cone (50%), (B) truncated cone (25%), (C) cone, and (D) paraboloid profiles. The three-dimensional simulation models of the structures that were used in this calculation are shown in the insets of Fig. 5. The corresponding structures were composed of a periodic pattern with a six-fold hexagonal symmetry for simplicity. As the height increased, the reflectance tended to decrease, and the low reflectance region was broadened toward longer wavelengths. These changes occurred because the effective refractive index changed more slowly. For the truncated cone, as the height increased from 0 to 500 nm, the reflectance oscillated in the 300–1400 nm wavelength region, indicating higher reflectance values of >1.5% (i.e., green parts). This oscillation was caused by interference between the upper and lower boundaries of the truncated cone, which acted as an effective medium (i.e., a single layer thin film with an abrupt change in the refractive index profile). However, the reflectance of the nanocones exhibit little dependence on height over a wide wavelength range of 300–1400 nm, thus indicating low reflectance values of <1% for heights greater than ~400 nm.

As shown in Fig. 5(D), the parabola-shaped SWS with a height of 300 nm had a broader AR spectral range (i.e., 300–1400 nm) than the cone-shaped SWS (300–900 nm) because the parabola-shaped structure provided a more linearly graded effective refractive index profile from air (n_air_ = 1) to fused silica than the cone-shaped structure. Thus, parabolic SWSs are more suitable for broadband AR than the conventional cone-shaped structures^31^. In addition, it is difficult to ensure an ideal nanocone shape formation because the SWS shape depends on control of the complex RIE process.

The optical properties of the SWS were easily controlled by varying their height and period, allowing the optics to be tailored to the required application. To obtain strong suppression of the reflectivity, the average SWS period (peak to peak length) must be controlled to an appropriate value. A large average period will result in light scattering and will influence the optical properties of the SWS surface. To create 0^th^ order grating, the minimal structure (i.e., period) should be less than the wavelength divided by the double refractive index of the material (*P* < *λ/2n*_*silica*_)^32^. However, the scattering on the disordered SWS was likely caused by Rayleigh scattering in our experiment. To suppress the scattering at the SWS surface, the average period should fulfil *P*≪*λ/*(*πn*_*silica*_)^33^. When the average period, P, was 100 nm, the calculated value of λ_min_ was ~458 nm. The calculated results agreed reasonably well with the experimental spectra, as indicated by the red line in Fig. 4(C). Figure S3 (B) shows diffuse scattering in the transmission direction, in the directional transmittance, and in the hemispherical transmission. Therefore, scattering in the transmission direction was the primary reason that the total efficiency was less than 100% in the short wavelength range.

**Figure 6:**
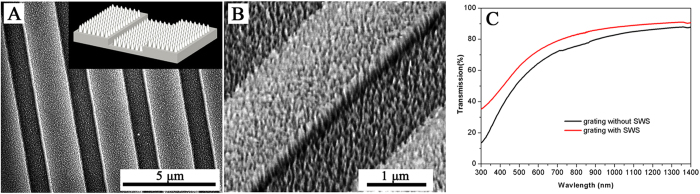
(**A**) Top view and (**B**) tilted view of the 3-μm-period grating that was coated with SWSs. The inset image is a schematic illustration of the fabricated SWS fused silica grating. (**C**) Measured transmission of the fabricated SWS fused silica grating at normal incidence.

To demonstrate the adaptability of this technology, we fabricated SWS on the grating surface with a period of 3 μm, a height of 200 nm and a duty cycle of 0.5. The surfaces of the ridges and grooves of each grating were covered with SWSs, as illustrated in Fig. 6(A,B). For the single-sided SWSs on the grating, the transmittance spectrum revealed a large improvement for the 300–1400 nm wavelength range, as shown in Fig. 6(C). High transmittance and diffraction efficiency were achieved due to the graded refractive index at the interface, which depended on the nanocone structure profile on the grating surface. The total reflectance of the fused silica grating was reduced from ~7.5% to ~4.5% (for the 550–700 nm wavelength range) due to the presence of SWSs on the grating surface (Figure S4). The reflected efficiencies of the 0^th^ and ±1^st^ diffracted orders for the fused silica grating with and without SWS were measured, as shown in Table S1. The reflected diffraction efficiencies were suppressed by the SWSs on the fused silica grating.

**Figure 7:**
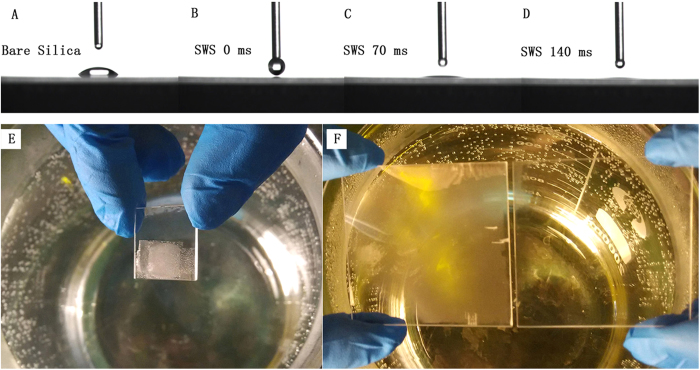
Superhydrophilic SWS surfaces on the fused silica substrate. (**A**) Water drop profile on a bare fused silica substrate. (**B**–**D**) Water drop profile on an SWS surface. (**E**) Comparison of fogging on the superhydrophilic SWS area (top) and the bare area (bottom). (**F**) The large scale anti-fogging SWS fused silica (right) and the bare fused silica (left). Figures (**E**,**F**) were obtained by X. Ye and L. Sun.

The fused silica SWSs significantly enhanced the surface hydrophilicity by inducing surface roughness on the hydrophilic fused silica substrate. The wettability of the covered SWSs and bare fused silica substrate surfaces was characterized via the water contact angle measurement method using a 3-μL water droplet at room temperature. Figure 7(A–D) shows the water droplet profile of the bare fused silica substrate and the fabricated fused silica SWS surfaces. The measured apparent water contact angle was ca. 25° for the bare fused silica substrate (Fig. 7A). When the water droplet contacted the fused silica SWS surface, it completely covered the surface within 0.14 s, and the apparent contact angle was ~0°. These results suggest that a superhydrophilic surface was achieved (Fig. 7B–D).

The evolution of the contact angle can be interpreted using Wenzel’s equation^34^ (cos θ_c_ = r cos θ), where θ is the intrinsic contact angle on the fused silica substrate, θ_c_ is the contact angle on the SWS surfaces, and r is the SWS surface roughness. Roughness is an important parameter of surface contact angles that can enhance the intrinsic wetting characteristics of the surface, as described by Wenzel’s equation. The surface roughness, r, increased due to the surface SWSs. Therefore, the obtained fused silica SWSs possessed a significant superhydrophilic property, leading to the anti-fogging property of the surface.

Superhydrophilic surfaces can dramatically suppress fogging behavior because condensation spreads rapidly on the surface, preventing light scattering from nucleated droplets. The anti-fogging experiments were performed by comparing fused silica with and without SWSs held above a vigorously boiling water bath. In Fig. 7(E), the upper half of the substrate, which was coated with SWSs on both sides, did not fog due to its superhydrophilic property. However, a large number of tiny condensed droplets appeared on the bottom half that was not coated with SWSs. Figure 7(F) shows that the bare fused silica substrate (left) was blurred by fog. However, the large fused silica substrate with the SWS coating on the surface maintained good transparency (right).

## Conclusions

We demonstrated a self-masking, one-step RIE process for creating high-performance AR and antifogging fused silica SWS. The fabricated SWSs on fused silica with an average structural period of ~100 nm exhibited excellent broadband AR and pronounced superhydrophilic properties. The self-masking RIE method is a fast, low cost, and scalable approach for fabricating large-scale AR structures. Furthermore, this method is applicable for the fabrication of diffraction gratingss with SWSs for reflection suppression.

## Materials and Methods

### SWS Fabrication

The fused silica SWSs were prepared using fluorine radical plasma etching in an RIE system. First, the RIE chamber was cleaned with O_2_ plasma. Prior to etching, the fused silica samples were cleaned using piranha solution (7:3 concentrated H_2_SO_4_:30% H_2_O_2_) for 2 h at 90 °C, followed by repeated rinsing with deionized water (18.2 MΩ cm^−1^). Then, the substrates were dried with a nitrogen stream and transferred to the RIE chamber. The gas reactants that were used for RIE included trifluoromethane (CHF_3_), sulfur hexafluoride (SF_6_), and helium (He). During etching, the radio frequency (RF) power was set to 100 W, and the chamber pressure was kept at ~200 mTorr. To avoid sputtering deposition of the cathode materials, the sample stage and the cathode were covered by a 6-inch silicon wafer during the RIE process. Then, the samples were cleaned using O_2_ plasma for 5 min.

### Characterization

The morphology of the SWSs was analyzed by scanning electron microscopy (SEM, ZEISS FESEM ULTRA55 and FEI ESEM Quanta 250). The planar fused silica substrate with the SWSs was sputtered with a thin layer of Au before imaging. The grating with the SWSs was measured using a FEI ESEM Quanta 250. The XPS measurements were performed using a Thermo ESCALAB 250. The monochromatized Al Ka source (1486.6 eV) was operated at a power of 150 W. During acquisition, the pass energy was set to 30 eV, and the spot size was 0.5 mm. The wavelength-dependent reflectance and transmittance measurements for wavelengths between 300 nm and 1400 nm were measured using a PerkinElmer Lambda 950 UV-VIS-NIR spectrophotometer. The transmittance spectrum was measured at an incidence angle of 0–45°, and the hemispherical total reflectance was measured with an integrating sphere. The water contact angles were measured using a JC2000C contact angle goniometer (Shanghai Zhongchen Digital Technique Apparatus). A deionized water droplet with a volume of 3 μL was placed on the surfaces to characterize wettability. The spreading of the water droplet on the surface of the SWSs was recorded using a high-speed CCD camera.

### Numerical Calculations

Numerical calculations were performed using the FDTD Solutions program purchased from Lumerical Solutions, Inc. (Vancouver, Canada). The incidence of the optical source was set for plane waves. The periodic boundary conditions were set around the unit cell, and perfectly matched layer (PML) absorbing boundary conditions were used at the top and bottom boundaries of the cell. The unit cell was designed in rectangle lattices (D * √3 D), where D is the diameter of the nanostructures, as shown in Figure S5.

## Additional Information

**How to cite this article**: Ye, X. *et al.* Formation of broadband antireflective and superhydrophilic subwavelength structures on fused silica using one-step self-masking reactive ion etching. *Sci. Rep.* **5**, 13023; doi: 10.1038/srep13023 (2015).

## Supplementary Material

Supplementary Information

## Figures and Tables

**Table 1:** Experimental conditions for the self-masking RIE process.

No.	Power(W)	Time(min)	CHF_3_(sccm)	SF_6_(sccm)	He(sccm)	Pressure(mTorr)
S1	100	20	25	10	100	200
S2	100	20	30	10	100	200
S3	100	20	40	10	100	200
S4	100	30	30	10	100	200
S5	100	40	30	10	100	200
