# Supplementary material for: Formation of broadband antireflective and superhydrophilic subwavelength structures on fused silica using one-step self-masking reactive ion etching
Source: Sci Rep. 2015 Aug 13;5:13023. doi: 10.1038/srep13023 (PMC4542686; doi:10.1038/srep13023)
Supplement: Supplementary Information [file srep13023-s1.doc]

**Formation of broadband antireflective and superhydrophilic subwavelength structures on fused silica using one-step self-masking reactive ion etching [[1]](#footnote-2) **

Xin Ye^1^, Xiaodong Jiang^1*^, Jin Huang^1^, Feng Geng^1^, Laixi Sun^1^, Xiaotao Zu^2^, Weidong Wu^1^ and Wanguo Zheng^1*^

^1^Research Center of Laser Fusion, China Academy of Engineering Physics, Mianyang, 621900, (P.R. China)

^2^School of Physical Electronics, University of Electronic Science and Technology of China, Chengdu, 610054, (P.R. China)

Figure S1. XPS (X-ray photoelectron spectroscopy) measurements of (A) self-masking RIE sample and (B) conventional RIE sample.

Figure S2. The tilt view SEM images of SWS on one side (A) and the other side (B) of double-sided sample.

Figure S3 (A) the reflectance spectrum including the specular, scattering, and total reflection. (B) The transmittance spectrum including the directional transmittance, hemispherical transmission and scattering in transmission direction.

Figure S4. Hemispherical reflectance of the fused silica grating with and without SWS.

Figure S5. The unit cell of numerical calculation was designed in rectangle lattices (D * √3 D), where D is diameter of nanostructure.

Table S1. Measured reflected efficiencies of the 0^th^ and ±1st diffracted orders for the fused silica with and without SWS for 30^o^ incident wavelengths of 405 and 671 nm. Reflection loss from the second interface isn’t included.

Figure S1


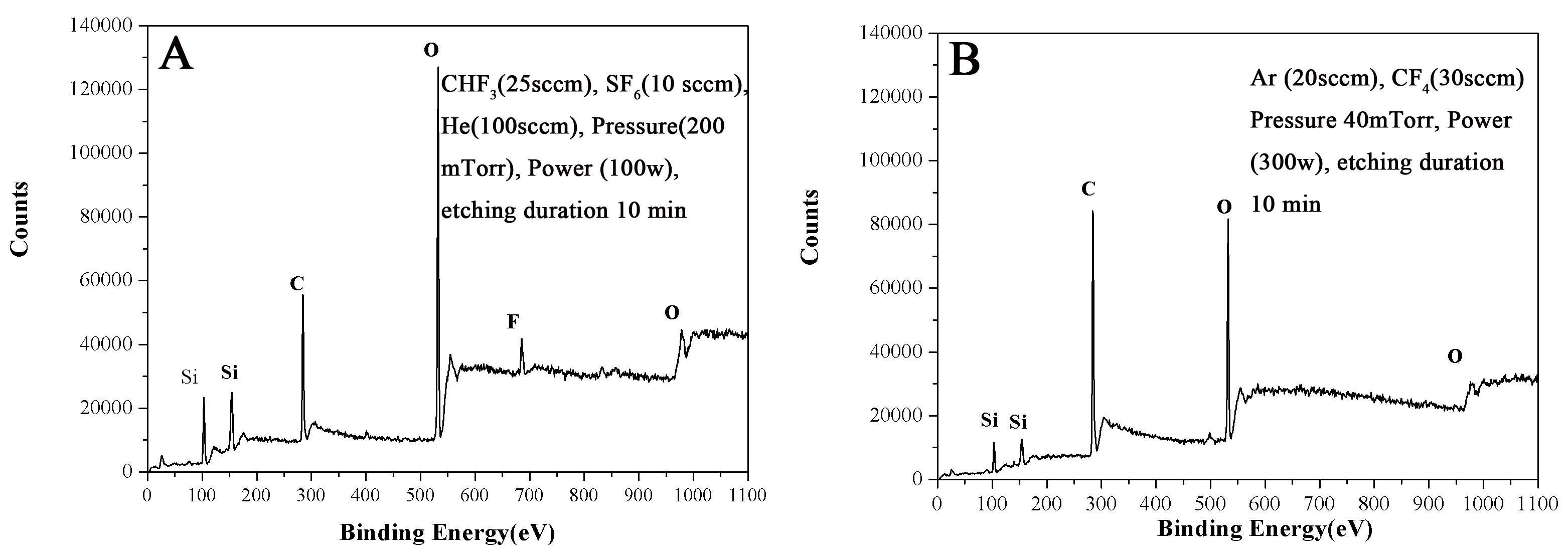


Figure S2


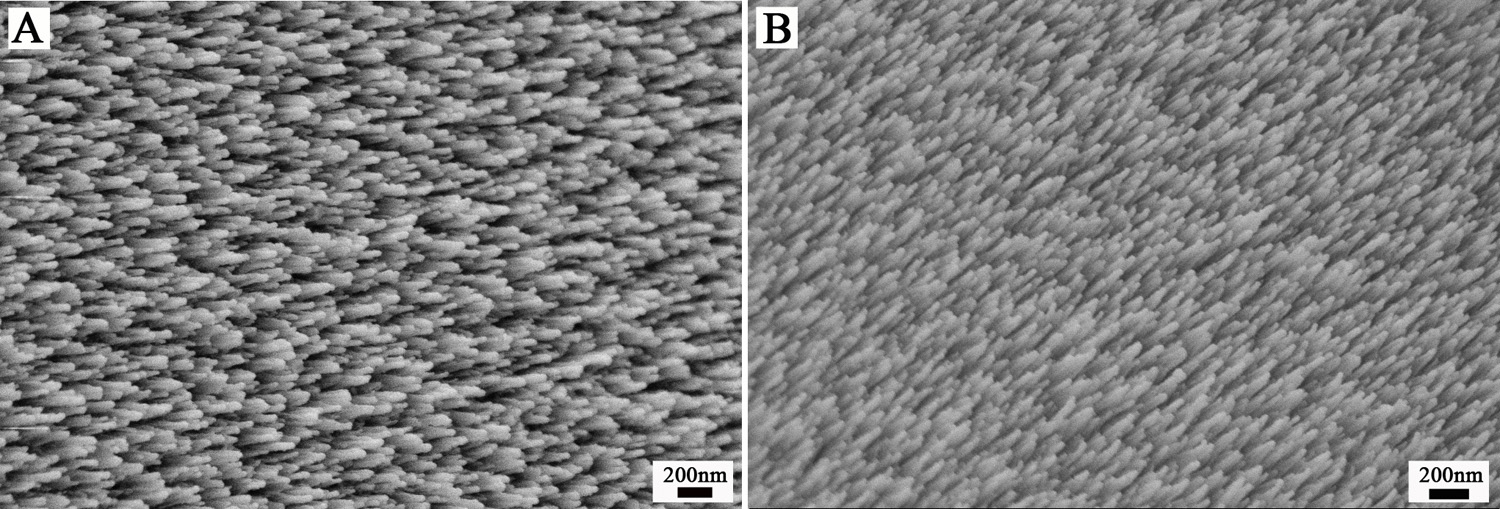


Figure S3


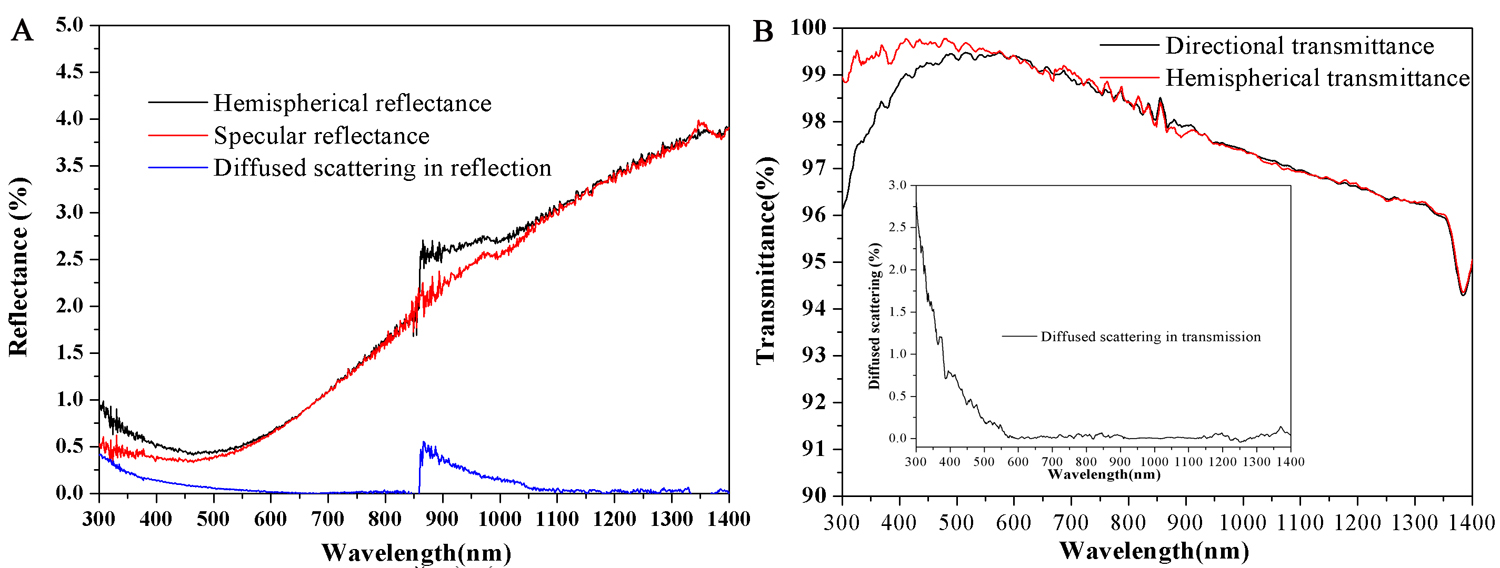


Figure S4


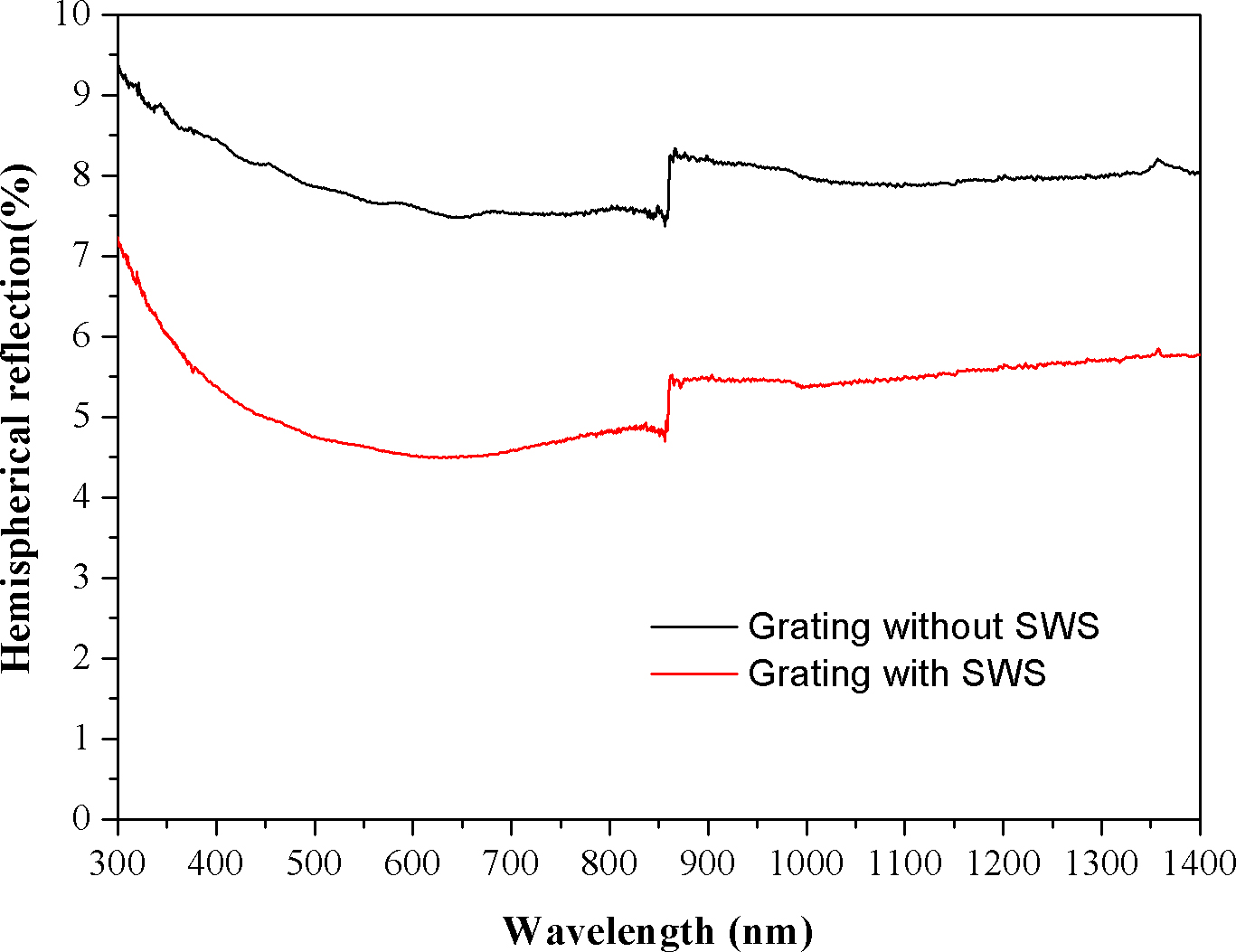


Figure S5


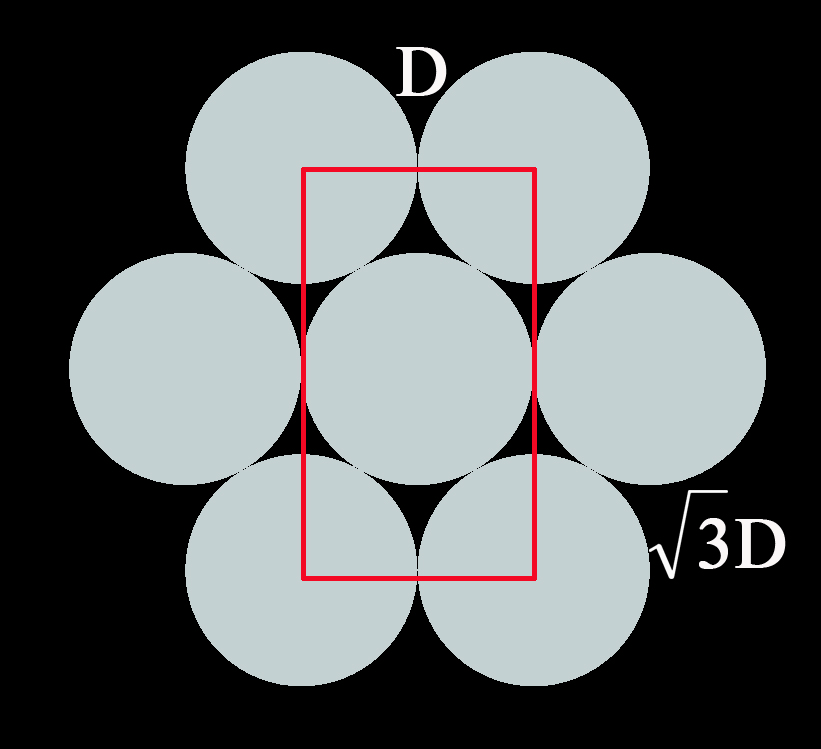


Table S1

| Wavelength | Order | Grating without SWS | Grating with SWS |
| --- | --- | --- | --- |
| 405nm | -1^st^ | 0.17% | 0 |
|  | 0^th^ | 2.04% | 0.16% |
|  | +1^st^ | 0.18% | 0 |
| 671nm | -1^st^ | 0.19% | 0 |
|  | 0^th^ | 1.67% | 0 |
|  | +1^st^ | 0.18% | 0 |

1.  * Correspondence should be addressed to X. D. Jiang ([jiangxdong@163.com](mailto:jiangxdong@163.com)) and W. G. Zheng (wgzheng_caep@sina.com)

   Tel: 86-0816-2480830; Fax: 86-0816-2480830 [↑](#footnote-ref-2)
